# Sociotechnical Needs of Registered Nurses in the Heart Failure Hospitalizations of African American Patients: Cross-Sectional Study

**DOI:** 10.2196/75080

**Published:** 2025-12-12

**Authors:** Tremaine Brueon Williams, Milan Bimali, Maryam Y Garza, Pearman Parker, Chase Paladino-Vaden, Emel Seker, Alisha Crump, Randy Rice, Latrina Prince, Taren Massey-Swindle, Kevin Wayne Sexton

**Affiliations:** 1 Department of Biomedical Informatics, College of Medicine, University of Arkansas for Medical Sciences, 4301 West Markham, Little Rock, AR, 72223, United States, 1 501 603 1766; 2 Department of Biostatistics, University of Arkansas for Medical Sciences, Little Rock, AR, United States; 3 Department of Population Health Sciences, The University of Texas Health Science Center at San Antonio, San Antonio, TX, United States; 4 College of Nursing, University of Arkansas for Medical Sciences, Little Rock, AR, United States; 5 Cardiovascular Service Line, Integrated Clinical Enterprise, University of Arkansas for Medical Sciences, Little Rock, AR, United States; 6 Graduate School, University of Arkansas for Medical Sciences, Little Rock, AR, United States; 7 Department of Pediatrics, College of Medicine, University of Arkansas for Medical Sciences, Little Rock, AR, United States; 8 Arkansas Children's Nutrition Center, Little Rock, AR, United States; 9 Department of Surgery, Vanderbilt University, Nashville, TN, United States; 10 Department of Biomedical Informatics, Vanderbilt University, Nashville, TN, United States

**Keywords:** socio-technical, human factors, EHR, registered nurses, health disparities, heart failure, electronic health record

## Abstract

**Background:**

The African American population is disproportionately impacted by congestive heart failure (CHF). The impact includes a hospitalization rate that is 2.5 times higher and a hospital stay that is, on average, a quarter of a day longer compared with Caucasians. Notably, nursing care has been associated with nearly a 30% decrease in hospitalizations and readmissions. Previous studies have demonstrated that registered nurses (RNs), working in conjunction with electronic health record systems to conduct care tasks, may optimize length of stay in African Americans with CHF.

**Objective:**

This study aimed to identify the needs of RNs who performed sociotechnical tasks, the perceived importance of these sociotechnical tasks, and the perceived performance of these tasks by RNs, in relation to the length of stay of their African American patients with CHF.

**Methods:**

The study used an observational, cross-sectional survey design in RNs who were randomly selected from a total population of 3498 RNs who provided care to 22,703 African American patients with CHF within 113,543 heart failure hospitalizations between January 1, 2015, and January 1, 2024. The RNs were retrospectively stratified into 2 groups based on EHR data: those whose African American patients had a mean length of stay of 10 days or less (Group A) and those whose mean length of stay was greater than 10 days (Group B). Descriptive statistics, Cohen *d*, and a 2-sided unpaired *t* test were used to analyze the data.

**Results:**

The total sample of 200 RNs responded to the survey (100% survey completion rate). Group A (100 RNs) reported the least important task as drawing conclusions about how to use the EHR to care for African American patients (mean 4.66, SD 1.82). The least important task in Group B (100 RNs) was reading published research on African American patients (mean 4.88, SD 1.70). Group A reported performing best in caring for African American patients (mean 5.61, SD 1.44). Group B reported performing best at caring for all patients (mean 5.86, SD 1.04). A total of 17 significant sociotechnical needs were identified among groups. In total, 2 sociotechnical needs were unique to group B: caring for patients (ie, the full scope of social and technological processes in nursing care; Cohen *d*=0.32, 95% CI 0.04-0.59; *P*=.04) and working with information related to a patient’s CHF in the EHR (eg, laboratory results, discharge summaries, or radiographic images) to care for the patient (Cohen *d*=0.33, 95% CI 0.05-0.61; *P*=.03).

**Conclusions:**

Lengths of patient stay may be reduced by identifying and addressing sociotechnical needs through targeted training, nursing care interventions, and RN-led risk stratification guidelines for working with EHRs to reduce lengths of stay in those who are disproportionately impacted by CHF.

## Introduction

According to the American Heart Association’s (AHA) 2024 heart disease and stroke update, nearly 6.7 million adults in the United States have congestive heart failure (CHF), a 12% increase since 2018 [1]. The prevalence of CHF is on track to reach the projected 46% increase from 2012 to 2030, affecting more than 8 million Americans [1]. African American men and women have the highest CHF-related, age-adjusted death rate, compared to other races [2,3] African American patients have an increased CHF hospitalization rate of nearly 2.5 times greater than Caucasians and a quarter of a day longer length of hospital stay, which are directly impacted by a substantial number of patient, provider, and social determinants of health factors that constrain guideline-directed medical therapy [1,3-5]. These longer lengths of stay increase the likelihood that African American patients experience iatrogenic complications that worsen their state of health [6,7]. Therefore, clinicians seek to optimize lengths of stay.

Tailored clinical care through guideline-directed medical therapy and specialized workflows for procedural treatments are innovative approaches to addressing hospitalization rates [8-11]. The AHA, American College of Cardiology, and the Heart Failure Society of America issued the 2022 joint AHA, American College of Cardiology, and Heart Failure Society of America guideline for the management of heart failure [9]. The guideline is the most recent directive for the design of future research on the effectiveness of nonclinical strategies directly related to hospitalization outcomes, such as assessing the efficacy and safety of including hydralazine isosorbide in the guideline-directed medical therapy of African American patients [9]. The guideline also included nonclinical strategies such as “addressing evidence gaps in women, racial, and ethnic populations” and “identifying the characteristics of systems of care (eg, disciplines and staffing, electronic health records (EHR), and models of care) that optimize guideline-directed medical therapy” [9].

Within systems of care, the use of multidisciplinary care teams that include cardiologists, advanced nurse practitioners, registered nurses (RNs), social workers, and dietitians has been shown to optimize guideline-directed medical therapy [9]. Multidisciplinary care teams with high levels of engagement from RNs have demonstrated the strongest associations with improved outcomes in African American patients with CHF [12,13]. The care provided by RNs within these teams has been associated with a 30% decrease in hospitalizations and a 31% decrease in readmissions over a 7-year period [12,13]. However, evidence of their effect on African American patients with CHF has been limited to simply assessing how the presence or absence of RNs on care teams is associated with hospitalizations and length of stay [12-14]. The care tasks performed by RNs in CHF management and care coordination are expansive, ranging from escalating and de-escalating care during hospitalization to discharge planning and care transitions. These tasks also include the use of invasive monitoring tools such as pulmonary artery catheters to educate patients on CHF in areas such as managing sodium intake, monitoring weight, and recognizing early signs and symptoms of decompensation [15,16].

Previous studies have identified EHRs as conduits of high-quality care delivery [12-14,17]. Evidence suggests that RNs, working in conjunction with EHRs to conduct these care tasks, affect care and outcomes [18-21]. As a result, this care management increases adherence to guideline-directed medical therapy and care plans. However, variation in how RNs perform these care tasks may indirectly contribute to differences in lengths of stay, which has been more directly driven by heart failure severity and social determinants of health [8-14,22-24]. These care tasks are defined as sociotechnical tasks, which are composed of any combination of the social (eg, interactions between RNs and patients) and technical (eg, EHRs, patient data, information, and tools) components of nursing care [25,26]. Additional examples of sociotechnical tasks are provided by the most widely recognized conceptual framework in the domain, sociotechnical framework for health information technology by Meeks et al [25] and Sittig and Singh [26] (Table 1). The 8 dimensions within the framework are empirically supported and serve as a foundation for examining sociotechnical tasks in nursing care [26]. For example, the first dimension of the framework is hardware and software computing infrastructure, which focuses on how RNs interact with, use the EHR, and its risk-specific indices [26]. When these tasks are important in care, but are performed poorly, a sociotechnical need exists [27].

**Table 1.** In total, 8 dimensions of Sittig and Singh’s sociotechnical framework for health information technology [26].

Dimension	Dimension focus	Dimension components
Hardware and software computing infrastructure	Focuses on how RNs^a^ interact with and overall use of the EHR^b^ and risk-specific indices	Computers, monitors, printers, and devices used to access the EHR
Clinical content	Focuses on how RNs work with CHF^c^-related textual, numeric, and imaging data within the EHR	Structured and unstructured data, information, and knowledge stored in the EHR
Human-computer interface	Focuses on the accessibility aspects of the EHR and risk-specific tools within the EHR that RNs see, touch, or hear as they assess patient risk	EHR design, functionality, feature availability, and ergonomic alignment of the EHR interface
People	Focuses on the humans associated with all aspects of the design, development, implementation, and RNs’ use of the EHR	Software developers, IT teams, system configuration, EHR training personnel, other clinicians, and patients
Workflow and communication	Focuses on how an RN works within clinical processes to ensure a patient with CHF receives the care they need when they need it	Engagement, collaboration, and cohesion between individuals and teams, EHR communication and messaging tools, and the alignment of EHR features with processes in care delivery
Internal organizational features	Focuses on how an RN works with policies, procedures, and the organizational culture of stratifying risk with the EHR	Organizational structures, leadership, and resources; IT policies and procedures
External rules and regulations	Focuses on how an RN works with external laws and requirements that facilitate or constrain the design, development, implementation, use, and evaluation of the EHR	Federal, state, and local laws that regulate the use of the EHR
Measurement and monitoring	Focuses on how an RN evaluates the consequences of EHR use in stratifying risks	Continuous evaluation of EHR features and functions, the development of methods and metrics

a RN: registered nurse.

b EHR: electronic health record.

c CHF: congestive heart failure.

Sociotechnical needs represent specific opportunities for additional training, care delivery improvements, and other interventions related to patients, providers, or social determinants of health [25,26]. If needs are addressed, the quality of care may improve, and the lengths of stay of patients may be optimized. These sociotechnical needs, the importance of the tasks, and their performance in relation to length of stay have not been examined. Yet, 96% of nonfederal acute care hospitals in the United States have adopted a certified EHR [28]. RNs may be most effective in working with the EHR and the information contained within it to perform real-time, in-hospital assessments to reduce poor outcomes and prevent mortality and longer lengths of stay [12]. Therefore, the aim of this study was to identify the needs of RNs who performed sociotechnical tasks, the perceived importance of these sociotechnical tasks, and the perceived performance of these tasks by RNs, in relation to the length of stay of their African American patients with CHF.

## Methods

This study was designed and reported in accordance with the guidelines of the Checklist for Reporting Results of Internet E-Surveys (CHERRIES) checklist [29].

### Study Design

This was a single-site study that used an observational, cross-sectional survey design. The observational nature of the study used EHR data from the Arkansas Clinical Data Repository to retrospectively track a target population of 3498 RNs who provided care to 22,703 patients with CHF within 113,543 heart failure hospitalizations between January 1, 2015, and January 1, 2024. The repository houses data extracted from the EPIC EHR and legacy systems and has been a secure source of data for other clinical and translational studies of the population [30]. A total of 200 RNs were randomly selected using stratified random sampling based on the mean length of stay of their African American patients. An adapted version of the Hennesey-Hicks Needs Analysis survey was administered to all RNs in the sample. Descriptive statistics, difference score, Cohen *d* coefficient, and a 2-sided unpaired *t* test were used to identify the perceived importance of these sociotechnical tasks, the perceived performance of these tasks by RNs, and their sociotechnical needs in relation to the length of stay of their African American patients with CHF.

### Ethical Considerations

The study procedures (protocol approval number #276211) were reviewed and approved by the University of Arkansas for Medical Sciences institutional review board. A dataset was provided to the study team by the repository that contained a deidentified list of patients and RNs, an edge list containing pseudonyms that linked patients with each RN who provided care during their hospitalization, health severity variables, and the length of stay of each African American patient’s hospitalization. A subsequent data request identified the names and email addresses of the 200 RNs who were surveyed. The RNs were provided with informed consent through email. The informed consent document embedded within the email included a basic description of the purpose of the study, the anticipated length of time survey completion, details on how data would be stored and accessed (where and how long), and contact information for the principal investigator. Data protection processes included not sharing individual responses outside of the study team, aggregating responses, segmenting access to identifying data and responses within the study team, storing data on only 1 university-encrypted laptop with multiple firewalls, and deleting identifying information after the study closed on June 30, 2025. After reviewing and agreeing to the informed consent presented through email, RNs were routed to the survey. A waiver of documentation of the informed consent process was granted by the institutional review board because the study presented only minimal risk for loss of confidentiality. The consent document is provided in Multimedia Appendix 1.

### Development and Pretesting

The survey, endorsed by the World Health Organization, is globally used within health care to assess task importance, performance, and needs of RNs and other clinicians [27]. In total, 33 previous studies of clinical practice have demonstrated the survey’s translation, cultural adaptation, and psychometric reliability in revealing the needs of RNs and other clinicians [27]. The standard survey contained 30 items consisting of basic care tasks [30]. After adaptation, a total of 22 items were in the final survey and represented the sociotechnical tasks performed by RNs during the provision of care. The survey instrument is provided in Multimedia Appendix 2.

Of the 22 final items, items 1‐14 were adapted from the basic items on the standard survey. The items were modified in alignment with the psychometric requirements of the survey manual [27]. For example, item number 1 of the standard survey required RNs to rate the following statement: “Establishing a relationship with patients.” The item was adapted for this study to state, “Using the EHR to establish a relationship with African American patients who have CHF.” The survey allowed for the removal and substitution of 8 of the standard items without compromising the psychometric properties (ie, reliability and validity) of the instrument. In total, 8 of the standard survey items were removed because they did not align with the objective of the study.

The remaining items, items 15‐22, were directly adapted from all 8 dimensions of the study’s conceptual framework, which is Sittig and Singh’s sociotechnical framework for health IT [26] (Table 1). The framework was chosen because it is the most comprehensive framework for understanding how RNs work with EHRs to improve patient outcomes.

The survey was pilot-tested for usability and technical functionality among an independent group of 5 RNs who reflected the sampling frame of the target population. The changes included increasing reminder intervals from monthly to biweekly, condensing introductory text for questions, and removing duplicate demographics data collection.

### Recruitment and Selection of RNs

The study was conducted on the main campus of an academic health center in a primarily rural state. The hospital has 535 beds, which generated the study data. Patients from all 75 counties of the state were cared for by RNs at the hospital. This included patients from counties with heart failure–related death rates that are twice the national average, of which more than 35% of residents lived in poverty [31-33].

A total of 3498 RNs were ranked numerically based on the mean length of stay of their patients with CHF. A sample of 200 RNs (100 per group; Group A and Group B) was randomly selected based on the mean length of stay of their patients with CHF being either above or below the mean of the total population of RNs (ie, 10 days). The RNs were not prospectively assigned to groups before they provided care to patients within the hospitalizations that generated lengths of stay. The RNs were retrospectively assigned to groups using a 10-day threshold based on whether their patients with CHF had short or long lengths of stay. Short and long lengths of stay did not distinguish between patients who received and did not receive optimal care. Optimal care was defined as the hospital’s provision of guideline-directed medical therapy [9] The 10-day threshold was selected because it represented the mean length of stay of patients with CHF in the total population of 3498 RNs. Additionally, patients with a length of stay of more than 10 days have a 52% higher risk of 30-day readmission and a 71% higher risk of mortality during 30 days of discharge, which could have confounded the length-of-stay outcomes [34]. Assuming a 2-sided type I error rate of 5%, with a sample size of 100 per group, the study was powered at 80% to detect an effect size of 0.29 or larger under the null hypothesis of zero effect size. The sample size estimates were obtained for effect size corresponding to sociotechnical needs using a one-sample *t* test and calculated using the proprietary PASS software [35].

**Figure 1.**
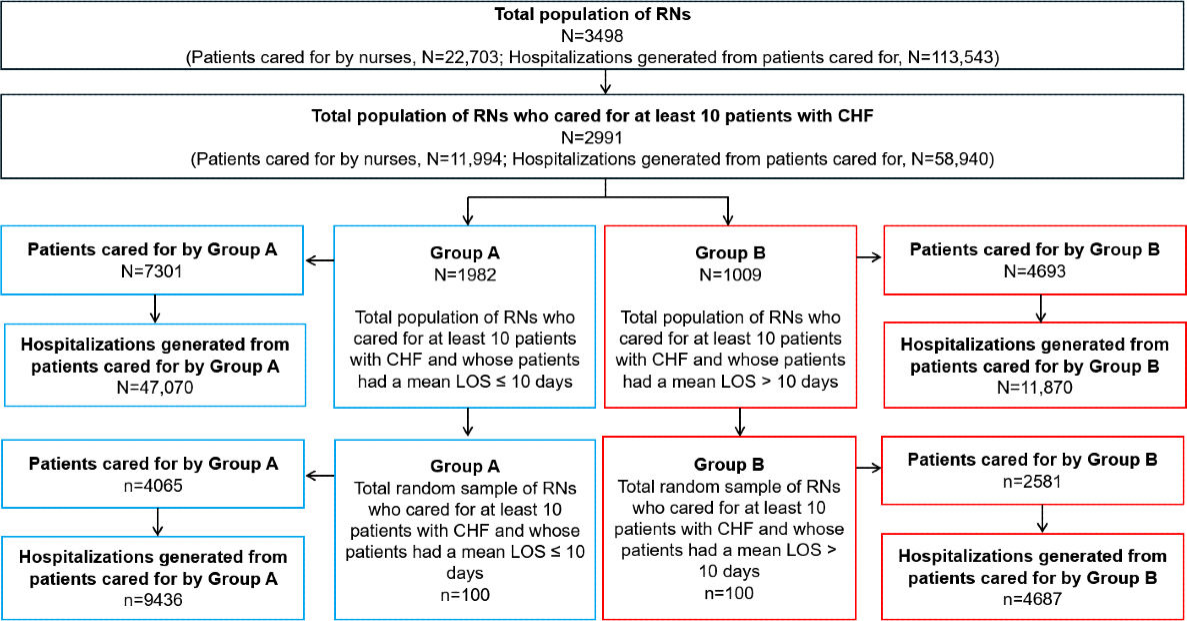
Flow diagram of registered nurse selection. CHF: congestive heart failure; LOS: length of stay; RN: registered nurse.

From the total population, a sample of 200 RNs was recruited and divided into 2 groups (Figure 1): Group A consisted of 100 RNs whose patients had a mean length of stay of 10 days or less (ie, patients with shorter lengths of stay), and Group B consisted of 100 RNs whose patients had a mean length of stay of more than 10 days (ie, patients with longer lengths of stay). RNs who cared for fewer than 10 patients with CHF were excluded from the selection to minimize the potential of selecting RNs who lacked sufficient experience in CHF care.

### Survey Administration

The survey was administered between November 15, 2024, and January 31, 2025. During the administration of the survey, RNs were instructed to reflect on their entire career of experiences working with EHRs to care for patients with CHF, not at any specific points in time. RNs were also instructed to reflect on their experiences working with all EHRs and not to focus on any specific EHR product or company.

Using a closed survey approach, all 200 RNs completed an electronic version of the same survey through the university-based, REDCap (Research Electronic Data Capture system; Vanderbilt University) [36,37]. Email addresses were loaded into the system and managed by one member of the study team for automated biweekly survey reminders and to prevent multiple entries from the same individual. Each RN received a custom survey link, which allowed the study team to determine completion.

The following demographic variables for the study were collected via the survey: sex, race, ethnicity, the number of years working as an RN, and the number of years working with patients who had chronic illnesses. None of the survey questions were mandatory, and the RNs could have ended the survey at any time.

The RNs manually rated all 22 survey items (sociotechnical tasks) on a 7-point Likert scale in 4 categories (Rating A-D). The RNs rated approximately 5 survey items on each of the 6 pages of the web-based survey in REDCap. The RNs were able to modify their ratings by toggling back and forth through the survey items before submission. Their ratings reflected how important the sociotechnical task was to their job (Rating A) and how well they were currently performing the sociotechnical task (Rating B). Higher ratings indicated greater perceived importance and perceived performance [27]. A sociotechnical need was defined as the difference between the performance and the importance of a task [27]. The sociotechnical need was the primary endpoint of the study. Similarly, the survey also assessed each group’s preferred approach to addressing any sociotechnical need through either care delivery intervention not related to training (Rating C) or through additional training alone (Rating D).

After completing the survey, RNs received a US $25 gift card for their participation. The offering of the gift card was disclosed to the RNs during the informed consent process.

### Other Variables

In total, 2 additional variables were provided within the data request to understand health severity in the patients cared for by both groups of RNs. The Van Walraven Elixhauser Comorbidity Score is a comorbidity-based measure of each patient’s overall health severity and is a valid and reliable predictor of in-hospital mortality [9,12,13]. Left ventricular ejection fraction, the percentage of blood pumped through the left ventricle of the heart, was captured via transthoracic echocardiogram during hospitalization and was used as a specific measure of heart function [9,12,13]. These variables have been established as measures of severity in African American patients with heart failure in the 2022 AHA, American College of Cardiology, or Heart Failure Society of America guideline for the management of heart failure and in previous studies [9,12,13].

### Bias

The selection of the survey tool as the study instrument minimized the potential for self-response bias because it has been psychometrically validated as providing valid and reliable information about task importance, performance, and needs. Stratified random sampling was applied to select the RNs from within each group. This improved the representation of the population in the sample, external validity, and the ability of the findings to be generalized to other states with similar characteristics (ie, rural, only one academic health center). Blinding was applied to reduce the potential influence of subjectivity in assessment and social desirability bias. The survey respondents and the study team members who administered the survey were blinded to group membership.

### Demographic Analysis

Descriptive statistics were presented using the mean and SD for continuous variables and frequencies and percentages for categorical variables. Descriptive statistics for demographic variables were presented for the overall sample and by study group.

### Analysis of Sociotechnical Task Importance, Performance, and Sociotechnical Need by Group

The sociotechnical need was calculated for each of the 22 sociotechnical items on the survey as the difference between overall mean responses to perceived importance (rating A) and perceived self-performance (rating B) [27]. A positive value indicated a need for a specific sociotechnical task (ie, high importance with low performance; the task was being performed at a level below its importance) [27]. A value of 0 indicated no need for a specific sociotechnical task (ie, the task was being performed at the same level of its importance) [27]. A negative value indicated no need for a specific sociotechnical task (ie, the task was being performed at a level above its importance) [27].

The difference score was tested for statistical significance using a 2-sided unpaired *t* test for each of the 22 survey items in each of the 2 study groups. To account for the multiplicity problem, we further obtained the false discovery rate–adjusted *P* values using Benjamini-Hochberg’s approach [38]. Statistical significance was determined using a false discovery rate–adjusted 2-sided *P* value threshold of .05, and uncertainty in parameter estimates was reported using a 2-sided 95% CI.

### Similarities and Differences in the Size of Sociotechnical Needs

The effect size of the difference score was estimated for each of the 22 tasks using the Cohen *d* coefficient [39]. The magnitude of effect size was determined using the following cutoffs that are (1) 0.20 for a small effect size, (2) 0.50 for a moderate effect size, (3) 0.08 for a large effect size, and (4) 1.30 for a very large effect size [39]. A forest plot was used to visualize the size of differences in sociotechnical needs by group.

### Similarities and Differences in the Rank of Priority of Significant Sociotechnical Needs

Cohen *d* values were ranked numerically within each group. A bump chart illustrated how each socio-technical need differed in priority, by group.

### Preferred Approach to Addressing Sociotechnical Needs by Group

The mean values of Rating C (addressing sociotechnical needs by addressing care delivery barriers that were not related to training) and Rating D (addressing sociotechnical needs through additional training, alone) were ranked numerically within each group for each of the 22 survey items. A higher mean in either rating indicated the preferred approach to addressing the sociotechnical need. A clustered bar chart was used to visualize the mean of each group’s preferred approach to any identified sociotechnical need.

## Results

### Overview

All 200 RNs completed the survey (100% survey completion rate), and there was no attrition. The 200 RNs provided care to 6646 African American patients with CHF within 14,123 heart failure hospitalizations between January 1, 2015, and January 1, 2024. All 200 RNs responded to all survey questions, and there were no missing data.

### Demographic Analysis Results

Based on survey data from the RNs, females were the largest sex, accounting for 82% (163/200, 82%) RNs in the overall sample (Table 2). Females were also the largest in each group, including 86% (86/100) from Group A and 77 (77/100) from Group B. Caucasian American patients were the largest racial group, accounting for 72% (144/200) of RNs in the overall sample, in Group A 72% (72/100), and in Group B 72% (72/100). Non-Hispanic and non-Latino RNs were the largest ethnic group, accounting for 92% (184/200) in the overall sample, 91% (91/100) in Group A, and 93% (93/100) in Group B.

**Table 2.** Survey demographic characteristics of registered nurses by group.

Characteristics	Overall	Group A	Group B
Sex, n (%)			
Female	163 (82)	86 (86)	77 (77)
Male	34 (17)	12 (12)	22 (22)
Unknown	3 (1.5)	2 (2)	1 (1)
Race, n (%)			
Caucasian American	144 (72)	72 (72)	72 (72)
African American	30 (15)	13 (13)	17 (17)
Asian American	11 (6)	6 (6)	5 (5)
American Indian or Alaskan Native	2 (1)	1 (1)	1 (1)
Native Hawaiian or Other Pacific Islander	1 (0.5)	0 (0)	1 (1)
Other race	12 (6)	8 (8)	4 (4.0)
Ethnicity, n (%)			
Non-Hispanic or Latino	184 (92)	91 (91)	93 (93)
Hispanic or Latino	9 (4.5)	5 (5)	4 (4)
Unknown	7 (3.5)	4 (4)	3 (3)

Based on EHR data on the RNs, Group A provided care for more patients with CHF than Group B, as evidenced by their larger mean number of patients with CHF cared for, with 414 (SD 415) versus 223 (SD 165) patients, respectively (Table 3). Group A also had a larger number of years of experience working as an RN than RNs who had patients with a mean length of stay of more than 10 days (Group B), with means of 11.97 (SD 7.97) versus 9.40 (SD 7.39), respectively. Group A had a larger number of years of experience working with patients who had chronic illnesses than group B, with means of 11.29 (SD 7.92) versus 8.97 (SD 6.83), respectively. The mean Van Walraven Elixhauser Comorbidity Score of patients (overall health severity) was approximately the same in patients cared for by RNs in Group A and Group B, 18.37 (SD 12.39) and 18.76 (SD 12.36), respectively. The mean left ventricular ejection fraction of patients (heart failure severity) was also approximately the same in patients cared for by RNs in Group A and Group B, 50.13 (SD 14.78) and 50.09 (SD 14.54), respectively.

**Table 3.** Electronic health record characteristics of the registered nurses and their patients by group.

Characteristics	Mean (SD)	Median (IQR)	Range
Number of patients with CHF^a^ cared for as an RN^b^			
Overall	319 (329)	218 (327)	10-1995
Group A	414 (415)	284 (381)	10-1995
Group B	223 (165)	164 (233)	10-840
Number of years working as an RN			
Overall	10.68 (7.78)	8 (9)	1.50-38
Group A	11.97 (7.97)	10 (10.25)	1.50-38
Group B	9.40 (7.39)	7 (7.50)	1.50-35
Number of years working with patients who had chronic illnesses as a registered nurse			
Overall	10.13 (7.46)	8 (8.25)	0-38
Group A	11.29 (7.92)	8.50 (9)	0-38
Group B	8.97 (6.83)	7 (7.50)	1.50-35
Patient length of stay (days)			
Overall	9.77 (3.19)	9.99 (4.13)	1.68-18.24
Group A	7.28 (2.37)	8.21 (2.74)	1.68-9.97
Group B	12.27 (1.50)	12.32 (1.73)	10.01-18.24
Patient Van Walraven Elixhauser Comorbidity Score (health severity)			
Overall	18.50 (12.37)	17 (17)	–14 to 66
Group A	18.37 (12.39)	17 (17)	–14 to 66
Group B	18.76 (12.36)	18 (16)	–11 to 66
Left ventricular ejection fraction (heart failure severity)			
Overall	50.12 (14.70)	55 (20)	5-80
Group A	50.13 (14.78)	55 (20)	5-80
Group B	50.09 (14.54)	55 (20)	10-75

a CHF: congestive heart failure.

b RN: registered nurse.

### Sociotechnical Task Importance Results

As shown in Table S1 in Multimedia Appendix 3, RNs in both groups rated all 22 tasks as having a high level of importance (>4) in improving the outcomes of African American patients with CHF. In both groups, the most important task was managing the overall workload of patients with the EHR (item 14; Group A: mean 6.36, SD 1.17; Group B: mean 6.63, SD 0.71). In RNs who had patients with a mean length of stay of 10 days or less, the least important task was drawing conclusions about how to use the EHR to care for African American patients (item 6; 4.66, SD 1.82). In RNs who had patients with a mean length of stay of more than 10 days, the least important task was reading published research on African American patients with CHF (mean 4.88, SD 1.70).

### Sociotechnical Task Performance Results

Table S1 in Multimedia Appendix 3 also presents results on task performance. In RNs who had patients with a mean length of stay of 10 days or less, the task that was performed the best was caring for African American patients with CHF (item 4; mean 5.61, SD 1.44). In RNs who had patients with a mean length of stay of greater than 10 days, the task that was performed the best was caring for all patients with CHF (item 3; mean 5.86, SD 1.04). In both groups, the task that was performed the poorest was reading published research on African American patients with CHF (item 2; Group A: mean 3.90, SD 1.96; Group B: mean 3.83, SD 1.78).

### Significant Sociotechnical Need Results

Sociotechnical needs were identified in 17 of the 22 survey items as evidenced by a statistically significant difference (false discovery rate–adjusted *P* values <.05) between RN ratings of importance and performance (Table S2 in Multimedia Appendix 3). In both groups, there were no sociotechnical needs identified in 5 of the 22 tasks (items 1, 4, 6, 11, and 21: *P*>.05). In total, 13 of the 17 sociotechnical needs (items 2, 5, 7‐10, 12‐14, 18‐20, and 22) were the same in both groups.

**Figure 2.**
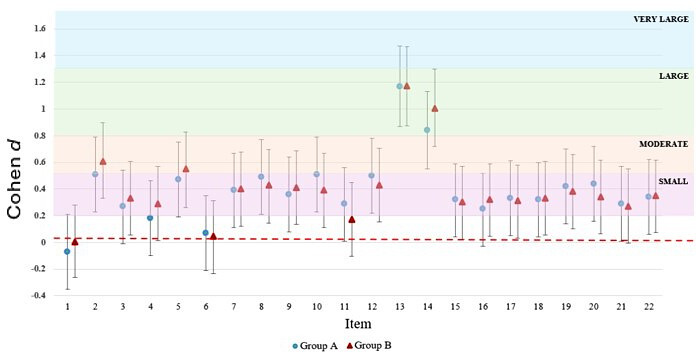
Forest plot of the size of significant sociotechnical need by group.

There were 2 additional sociotechnical needs unique to RNs who had patients with a mean length of stay of 10 days or less (items 15 and 17), as shown in Table S2 in Multimedia Appendix 3 and Figure 2. These needs were working with hardware and software related to the EHR to care for a patient with CHF (Cohen *d*=0.32, 95% CI 0.04-0.59; *P*=.04) and working with the design of the EHR (eg, parts of the EHR’s screens that you can see, touch, or hear) as they retrieved information to provide care to patients with CHF (Cohen *d*=0.33, 95% CI 0.05-0.61; *P*=.03).

There were also 2 additional sociotechnical needs unique to RNs who had patients with a mean length of stay of more than 10 days (items 3 and 16). These needs were caring for patients with CHF (Cohen *d*=0.32, 95% CI 0.04-0.60; *P*=.04) and working with information related to a patient’s CHF in the EHR (eg, laboratory results, discharge summaries, or radiographic images) to care for the patient (Cohen *d*=0.31, 95% CI 0.03-0.58; *P*=.047).

### Similarities in the Size of Significant Sociotechnical Needs Among Groups

Of the 13 sociotechnical needs that were common among both groups, 10 were similar in size. The 2 largest sociotechnical needs (item 13 and 14) were the same in both groups, as defined by a Cohen *d* of greater than or equal to 0.80 in both groups: personally coping with burnout in their clinical environment (Group A: Cohen *d*=1.17, 95% CI 0.87-1.47; *P*<.001 and Group B: Cohen *d*=1.17, 95% CI 0.87-1.47*; P*<.001) and managing their overall workload of patients with the EHR (Group A: Cohen *d*=0.84, 95% CI 0.55-1.13; *P*<.001 and Group B, Cohen *d*=1, 95% CI 0.71-1.30; *P*<.001). The only moderate-sized sociotechnical need that was the same in both groups was reading published research on African American patients with CHF (item 2: Group A Cohen *d*=0.51, 95% CI 0.23-0.79; *P*<.001 and Group B Cohen *d*=0.60, 95% CI 0.32-0.89; *P*<.001). Figure 2 presents 7 additional small training needs that were similar in size among groups (items 7‐9, 18‐20, and 22).

### Differences in the Size of Significant Sociotechnical Needs Between Each Group

Of the 13 sociotechnical needs that were common among groups, 3 sociotechnical needs differed in size between groups. Giving patient education information to African American patients or their caregivers (item 5) was a small need in RNs who had patients with a mean length of stay of 10 days or less, (Group A: Cohen *d*=0.47, 95% CI 0.19-0.75; *P*=.003), but a moderate need in RNs who had patients with a mean length of stay of more than 10 days (Group B, Cohen *d*=0.54, 95% CI 0.25-0.82; *P*<.001). Collecting relevant information on the social determinants of health (eg, education, health literacy, safe housing, and access to nutritious food) from the EHR (item 10) was a moderate need in RNs who had patients with a mean length of stay of 10 days or less (Group A: Cohen *d*=0.51, 95% CI 0.23-0.79; *P*<.001), but a small need in RNs who had patients with a mean length of stay of more than 10 days (Group B: Cohen *d*=0.38, 95% CI 0.1-0.66*; P*=.02). Accessing clinical resources to care for their patients with CHF (item 12) was a moderate sociotechnical need in RNs who had patients with a mean length of stay of 10 days or less (Group A, Cohen *d*=0.50, 95% CI 0.22-0.78; *P*=.003), but a small need in RNs who had patients with a mean length of stay of more than 10 days (Group B: Cohen *d*=0.42, 95% CI 0.14-0.70; *P*=.01).

### Similarities in the Rank of Priority of Significant Sociotechnical Needs in Each Group

The top 3 sociotechnical needs in both groups were the same (Figure 2): personally coping with burnout in their clinical environment (item number 13), managing their overall workload of patients with the EHR (item number 14), and reading published research on African American patients with CHF (item number 2), respectively. Accessing clinical resources to care for their patients with CHF (item number 12) was ranked fifth in both groups. Undertaking health promotion and prevention tasks to care for African American patients with CHF (item number 8) was ranked sixth in both groups. Within Figure 2, items with statistically significant sociotechnical needs (*P*<.05) are denoted by an asterisk.

### Differences in the Rank of Priority of Significant Sociotechnical Needs in Each Group

**Figure 3.**
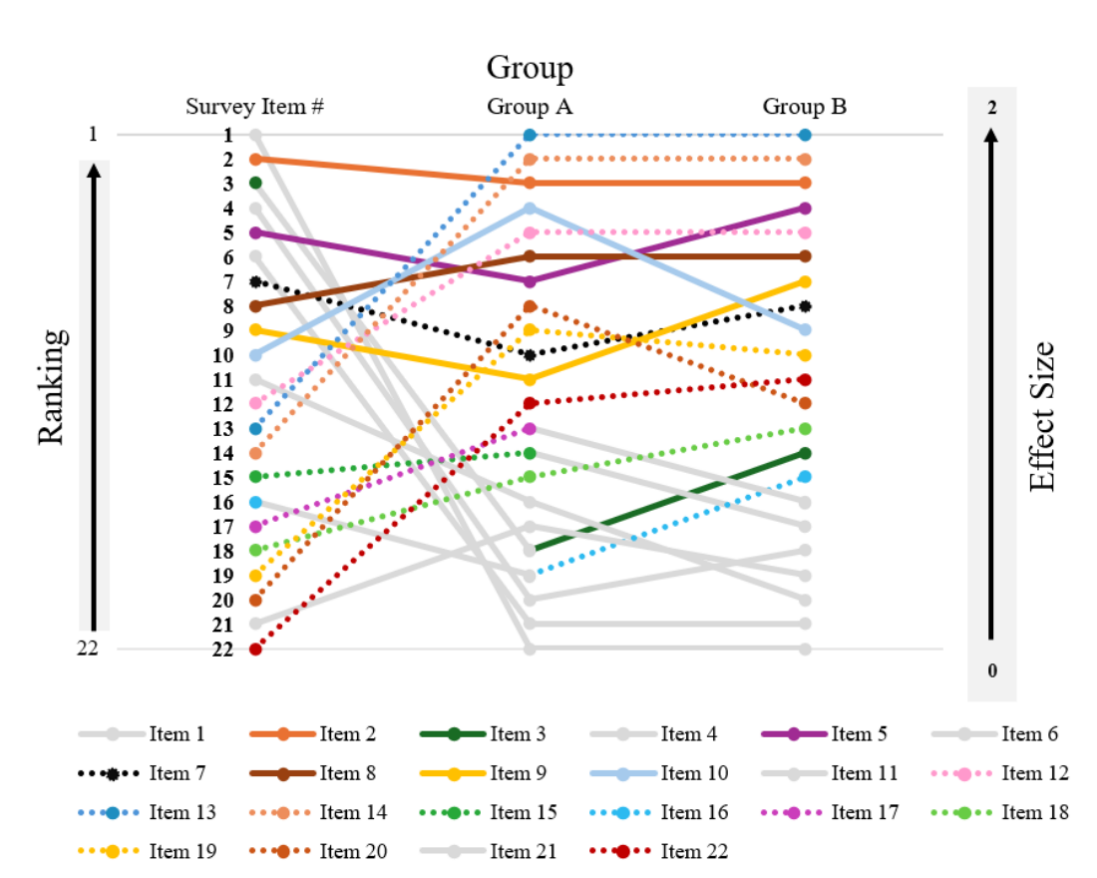
Cohen *d* ranking of significant sociotechnical needs by group.

Figure 3 ranked all 22 sociotechnical tasks in each group from greatest need (ie, 1) to the least (ie, 22) by Cohen *d*. Within Figure 3, items with no sociotechnical needs (*P*≥.05) were represented by solid gray lines and circles. The figure demonstrates how each of the significant sociotechnical needs changed in perceived priority in RNs who had patients with a mean length of stay of 10 days or less compared to those who had patients with a mean length of stay of more than 10 days.

Three items were ranked higher in RNs who had patients with a mean length of stay of 10 days and less than RNs who had patients with a mean length of stay of more than 10 days, including: collecting relevant information on the social determinants of health from the EHR (item 10; group A: 4th and group B: 9th), working within internal organizational policies, procedures, and culture related to the EHR to care for patients with CHF (item 20; Group A: 8th, and Group B: 12th), and using current processes to share information that provides each patient with CHF with the care they need at the time they need it (item 19; Group A: 9th and Group B: 10th).

In total, 5 items were ranked higher in RNs who had patients with a mean length of stay of more than 10 days than in RNs who had patients with a mean length of stay of 10 days and less, including (1) giving patient education information to African American patients or their caregivers (item 5; group A: 7th and group B: 4th), (2) using risk scores or other information from the EHR to improve a patient’s health (item 7; group A: 10th and group B: 8th), (3) assessing African American patients’ clinical needs using the EHR (item 9; group A: 11th and group B: 7th), (4) continuously evaluating the quality of care that results from their use of the EHR to provide care for patients with CHF (item 22; group A: 12th and group B: 11th), and (5) the training or performance of other people in their environment who use the EHR (item 18; group A: 15th and group B: 13th).

In total, 4 additional sociotechnical needs only ranked in one of the groups due to a lack of significance in the other group. Working with hardware and software related to the EHR to care for a patient with CHF (item number 15) ranked 14th among RNs who had patients with a mean length of stay of 10 days or less (Group A), but was not significant in RNs who had patients with a mean length of stay of more than 10 days (Group B). Working with information related to a patient’s CHF in the EHR to care for the patient (item 16) ranked 15th in RNs who had patients with a mean length of stay of more than 10 days (Group B), but was not significant in RNs who had patients with a mean length of stay of 10 days or less (Group A). Working with the design of the EHR to retrieve information to provide care to patients with CHF (item number 17) ranked 13th among RNs who had patients with a mean length of stay of 10 days or less (Group A), but was not significant in RNs who had patients with a mean length of stay of more than 10 days (Group B). Caring for patients with CHF (item 3) was ranked 14th in RNs who had patients with a mean length of stay of more than 10 days (Group B) but was not significant in RNs who had patients with a mean length of stay of 10 days or less (Group A).

### Preferred Training and Care Delivery Barriers by Group

**Figure 4.**
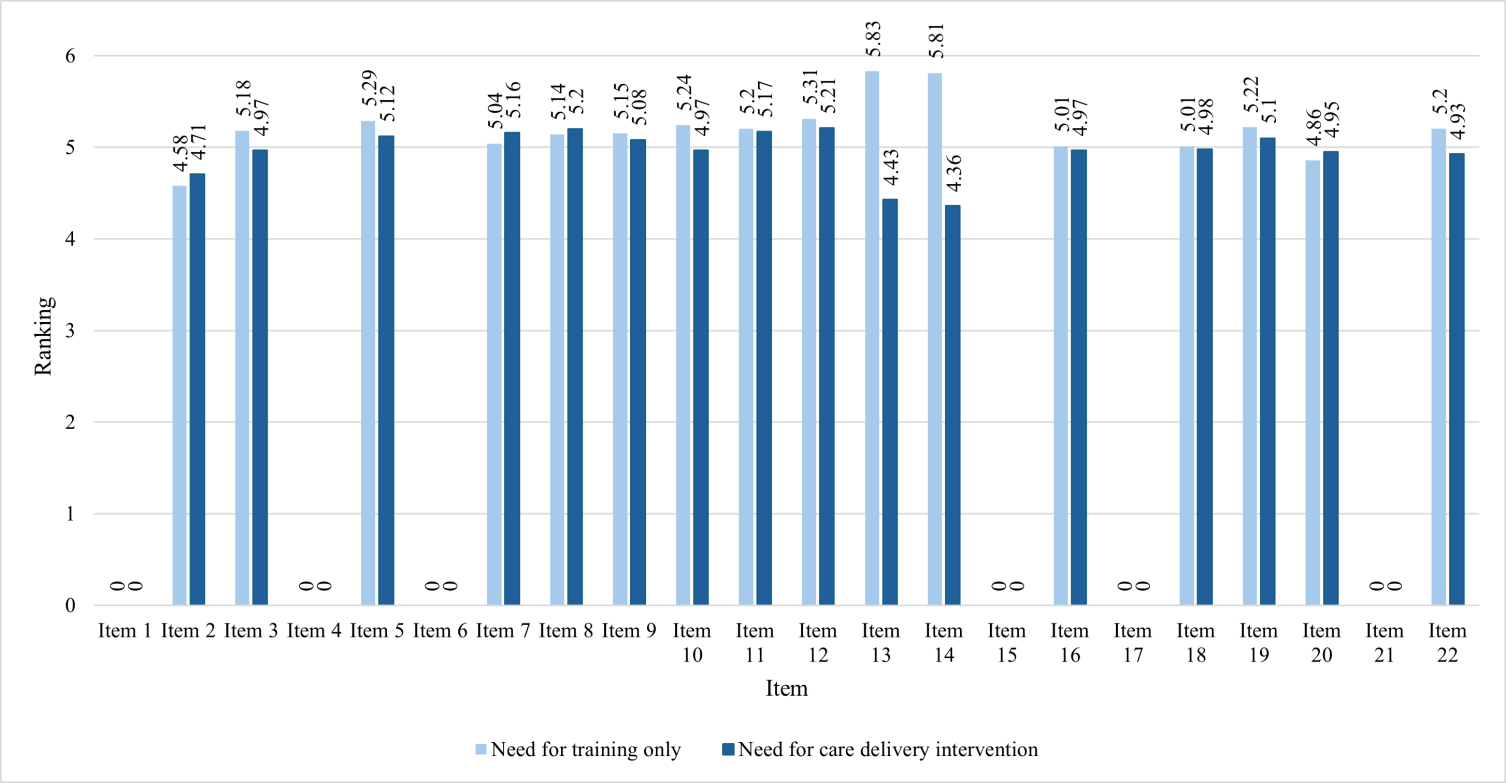
Preferred approaches for significant sociotechnical needs in Group A.

**Figure 5.**
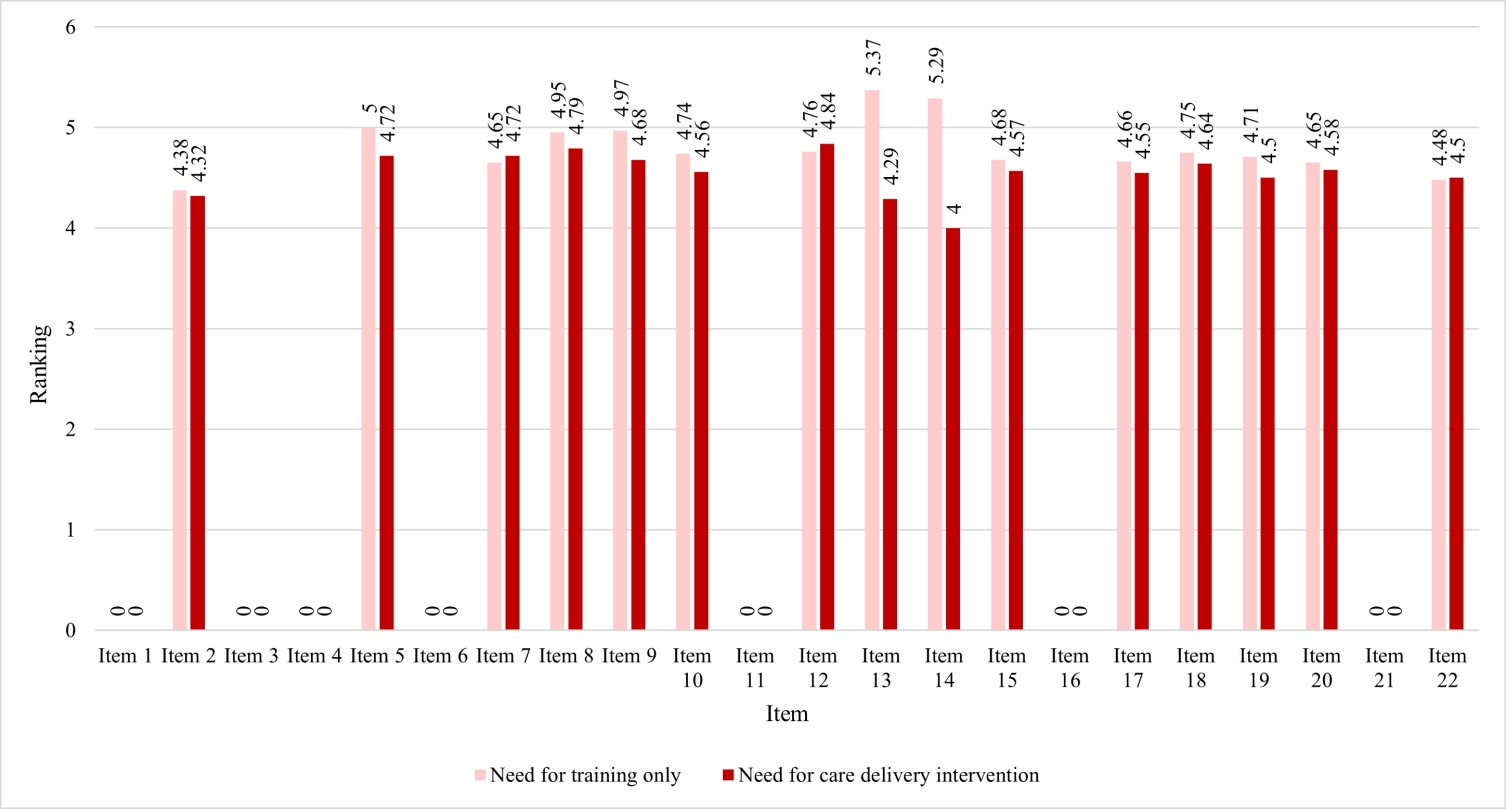
Preferred approaches for significant sociotechnical needs in Group B.

Figures4  5 present the mean ratings of the RNs’ preferred approaches to addressing the significant sociotechnical needs by addressing care delivery barriers that were not related to training (Rating C) or through additional training alone (Rating D). Higher ratings represent a preferred approach. For example, in using risk scores or other information from the EHR to improve a patient’s health (item 7), RNs who had patients with a mean length of stay of 10 days or less (Figure 4) and RNs who had patients with a mean length of stay of more than 10 days (Figure 5) preferred to have the sociotechnical need met by addressing care delivery barriers that were not related to training. RNs who had patients with a mean length of stay of 10 days or less (Group A) preferred training alone in 12 of the needs (items 2, 5, 8, 9, 10, 13, 14, 15, 17, 18, 19, and 20), while they preferred to have care delivery barriers addressed in 3 sociotechnical tasks (items 7, 12, and 22). RNs who had patients with a mean length of stay of more than 10 days (Group B) preferred training alone in 10 of the needs (items 3, 5, 9, 10, 12, 13, 14, 16, 19, and 22), while they preferred to have care delivery barriers addressed in 3 sociotechnical tasks (items 2, 7, 8, and 20).

## Discussion

### Overview

The study identified 17 sociotechnical needs of RNs that indicate specific opportunities for additional training, care delivery, EHR improvements, and other interventions related to patients, providers, and social determinants of health.

### Summary of Key Findings

In total, 2 sociotechnical needs were unique to RNs who had patients with long lengths of stay: working with information related to a patient’s CHF in the EHR and caring for patients with CHF. Giving patient education information to African American patients or their caregivers was an additional need that ranked as a higher priority in RNs who had patients with long lengths of stay, when compared to those who had patients with short lengths of stay. The 2 largest sociotechnical needs were the same in both groups and were related to RNs’ perceptions of their workload: managing their overall workload of patients with the EHR and personally coping with burnout in their clinical environment. Patients cared for by both groups of RNs had approximately the same levels of overall health severity and heart failure severity, indicating that differences in lengths of stay may not be solely influenced by these factors of a patient’s health severity. The finding also indicates that there may be other patient, provider, and structural factors, such as the high burden of social determinants of health on African Americans, that may be attributed to length of stay. Addressing these factors in addition to the sociotechnical needs of RNs could provide a more comprehensive and effective approach to reducing lengths of stay and mortality.

### The Potential for Reducing Long Lengths of Stay

One of the largest sociotechnical needs that was unique to RNs who had patients with long lengths of stay reflected the full scope of social and technological processes performed by RNs during care delivery, caring for patients with CHF. A similar sociotechnical need was ranked as a higher priority in RNs with long lengths of stay: assessing African American patients’ clinical needs using the EHR. These tasks are based on crucial assessments that include the active monitoring of symptoms, vital signs, fluid intake and output, and daily weight measurement [15]. The extent to which these assessments are performed is critical to the effective provision of care by other care team members [12,15]. These findings showed that the unaddressed sociotechnical needs of RNs may be associated with longer lengths of stay. RNs who had patients with long lengths of stay preferred to have these needs addressed by additional training, suggesting that care delivery barriers had a lesser influence on their performance or needs. Additional evidence-based training could be provided through the American Association of Heart Failure RNs’ Heart Failure Certification, which has standardized the specialized CHF knowledge base of RNs and regulates the clinical experiences of RNs in heart failure care [15].

Another sociotechnical need that was unique to RNs who had patients with long lengths of stay was working with information related to a patient’s CHF in the EHR to care for the patient. The EHR contains data from an array of laboratory tests (eg, troponin, basic metabolic panel, magnesium, hemoglobin A_1c_, urinalysis, lipid profile, liver function tests, and a complete blood count) [15]. These tests are used to distinguish between worsening CHF and symptoms such as shortness of breath that mimic other illnesses [15]. For example, laboratory tests containing information on B-type natriuretic peptide levels and pulmonary artery pressure are used as biological markers for monitoring heart function [15]. RNs review, use, and monitor these data to escalate and de-escalate care provided by other members of the care team [15,16]. Our findings suggest that long lengths of stay may be reduced through additional training in retrieving, using, and monitoring these data because these data and information are nationally recognized indicators of hospitalizations and widely used in remote monitoring [15,40,41].

Giving patient education information to African American patients or their caregivers was ranked as a higher priority by RNs who had patients with longer lengths of stay when compared to those with shorter lengths of stay. The size of the need was small in RNs with shorter lengths of stay, but a moderate-sized need in RNs with longer lengths of stay. These differences in the rank and size of the need could be related to the longer lengths of stay. For hospitalized African American patients with CHF, education information provided by the RN is the sole opportunity to review and discuss signs, symptoms, laboratory results, medications, dietary restrictions, fluid management, and follow-up care [15]. RNs educate patients in a manner that is more understandable than other care team members who may use more lengthy and complex medical jargon [15]. An RN’s ability to translate complex medical terminology is limited by the patient’s education and understanding of care components, such as the patient’s regimen, purpose, and dosing of heart failure medications [24]. The preferred approach to addressing this need was training. For example, this could include additional training on the RN’s retrieval of a medication synopsis from an optimized medication administration record. Training related to using this synopsis could be provided. This process may provide additional opportunities to expand the capacity of EHRs to support RNs who may use sources external to EHRs for tasks such as searching for medications on a one-by-one basis for patient education.

An RN’s ability to use this information to educate patients is also critical to patient self-management of their CHF, as the complex nature of CHF care requires multiple transitions between providers and frequent transitions between health care systems [16]. This initial education (and ongoing education) is critical to a patient’s ability to establish a baseline understanding of their CHF case and conduct any self-assessment of any deviations or worsening symptoms [16]. This education provides patients with the ability to more effectively communicate changes in their symptoms with various members of their multidisciplinary and specialty care teams. Patient education may be optimized in RNs who use evidence-based methods such as teach-back techniques, message repetition, role-playing, and clinic aids such as brochures and videos [16]. These techniques verify patient understanding, which could be highly effective in African Americans who are burdened by health literacy [3,16].

### Potential Contributions of the EHR and Its Data

EHR use is frequently not optimized in heart failure management [22]. Previous research on care transitions has shown that seamless postdischarge support from family, caregivers, and primary care providers optimizes outcomes and adherence to the plan of care [9,15,22,23]. The EHR can support care transition by addressing social determinants, such as clinician biases and resistance to evaluating African American patients for advanced heart failure therapies [9,15,16,23]. EHRs can also be optimized to identify daily discharges to support referrals for patients to cardiologists, as African American patients are also less likely to receive care from a cardiologist during a heart failure hospitalization [9]. Strategies such as automated reminders using push notifications in patient portals and among scheduling departments may optimize care transition. EHRs can also be optimized to provide referrals to home care agencies, palliative care, and longer-term services by facilitating high-quality, structured communication focused on shared decision-making [9,15,16]. The EHR could automate referrals when patients meet criteria or have a provider in the loop, alerting the system to let the primary team know that the patient meets criteria and force an active decision to refer or not refer to palliative care [15]. For longer-term care services, the EHR can facilitate notifications via case managers that assist with postacute discharge arrangements [15].

EHRs facilitate the storage and retrieval of standardized education materials, generally developed by reputable sources such as the AHA and professional associations [24]. Traditional approaches to CHF management have previously limited patient education to information based on the management of symptoms, weight, and vital signs, which require less education when compared to the more complex biomarkers produced by remote monitoring tools [15,40,41]. Contemporary approaches, such as the use of remote monitoring tools, facilitate real-time adjustments of care plans based on data collected through tools such as the CardioMEMS system [41]. The CardioMEMS system, for example, provides RNs with early notification of fluid build-up. The system identifies pressure and fluid build-up weeks before a potential hospitalization, compared to traditional approaches that rely on symptoms to develop [15]. Traditional approaches rely on the patient to be in the same physical location as the RN (ie, a hospitalization or clinic visit) or for the patients to capture their weight and vital signs, remotely, at regular intervals, and communicate changes in, for example, weight, to the RN [15,40,41]. This process requires physical tools and technologies such as scales, blood pressure cuffs, and pulse oximeters. It is not likely that the high rates of poverty in the African American communities that were cared for by the RNs allow patients to absorb the costs of these tools [31-33]. The use of these tools in both traditional and advanced CHF management approaches may optimize education toolkits to support the RNs in intervening by contacting the patient to identify and address the cause of the pressure and fluid build-up [40-42]. These data and information could be fed into the EHR manually or automatically through remote monitoring tools. This data could fuel EHR alerts to RNs and patients regarding significant changes and deviations from established thresholds for weight, heart rate, and blood pressure, which are early signs of fluid build-up and worsening heart function [40-42]. For patients, notifications from patient portals that acknowledge worsening heart function may encourage patients to seek out additional care instead of patients waiting for any number of care team members to reach out to them, which could increase the patient’s likelihood of more severe symptoms and readmission. These processes rely on the patient’s understanding of their CHF and their ability to communicate relevant information to the RN for effective triage. The significant reliance on people to understand, assess, and communicate information within this process is an opportunity for artificial intelligence algorithms to supplement this process. These algorithms could support the timely detection and communication of invisibles that are not recognized by patients and relieve some of the cognitive burden of complex sociotechnical tasks performed by RNs.

### Limitations

The study used an observational, cross-sectional study design, which could not infer that nursing care caused lengths of stay or that the EHR had a significant effect on length of stay. The study design found statistically significant and meaningful sociotechnical needs of RNs who had patients with lengths of stay of 10 days or less, and in RNs who had patients with lengths of stay of more than 10 days. Empirical evidence suggests that lengths of stay may be more directly associated with or caused by variables such as CHF severity, patient adherence to evidence-based therapies, self-management behaviors, and social determinants of health [9,12,13].

Identifying patterns in nursing care by retrospectively stratifying RNs into groups based on the mean length of stay of their patients was a broad approach to assessing nursing expertise, care priorities, and length of stay. Although patients cared for by both groups of RNs had relatively the same left ventricular ejection fraction and Van Walraven Elixhauser Comorbidity Score, it is possible that patients with a longer length of stay were sicker, as defined by other hemodynamic values. It may also be possible for patients with longer lengths of stay to have received inadequate medical care before admission, were less compliant to the prescribed plan of care, had significant social, psychological, or resource barriers (eg, health literacy, cognitive impairment, lack of caregiver support), or were more likely to be approaching end of life where even optimal care would have been futile. Controlling for these and other variables of severity in additional studies that use experimental designs will be most impactful in evaluating and inferring causation between the socio-technical tasks and patients’ length of stay.

Future studies should use specific evidence-based heart failure therapy guidelines to define optimal care, such as assessing the sociotechnical tasks in relation to groups whose African American patients were prescribed a combination of hydralazine and isosorbide dinitrate at discharge, compared to patients who were not. Defining optimal care based on heart failure guidelines that assess and address social determinants of health (eg, scheduling and coordinating transportation to follow-up visits within 7 days of discharge) may be most impactful.

The timing of the survey administration may not have accurately captured every sociotechnical need during each hospitalization of each patient because the sociotechnical needs of RNs may vary over time. It was not possible to administer the survey to every RN during each hospitalization without disrupting patient care and increasing the potential for acquiescence bias. To minimize the potential impact of this limitation, RNs were instructed to reflect on their entire career of experiences working with EHRs to care for patients with CHF, not at any specific point in time. To avoid bias toward or against any EHR, RNs were instructed to reflect on their entire career of experiences working with all EHRs and not to focus on a specific EHR or company.

### Conclusions

RNs who had African American patients with long lengths of stay had unique sociotechnical needs. Lengths of patient stay may be reduced by addressing these needs through training, nursing care, RN-centered EHR interventions, and RN-led risk stratification guidelines for working with EHRs to optimize lengths of stay in African American patients who are disproportionately impacted by CHF.

## Supplementary material

10.2196/75080 Multimedia Appendix 1 Informed consent email.

10.2196/75080 Multimedia Appendix 2 Survey instrument.

10.2196/75080 Multimedia Appendix 3 Tables for sociotechnical task importance, performance, and needs.

10.2196/75080 Checklist 1 CHERRIES Checklist.

## Abbreviations

AHA: American Heart Association
CHERRIES: Checklist for Reporting Results of Internet E-Surveys
CHF: congestive heart failure
EHR: electronic health record
REDCap: Research Electronic Data Capture
RN: registered nurse
