# Supplementary material for: Sociotechnical Needs of Registered Nurses in the Heart Failure Hospitalizations of African American Patients: Cross-Sectional Study
Source: JMIR Nurs. 2025 Dec 12;8:e75080. doi: 10.2196/75080 (PMC12700336; doi:10.2196/75080)
Supplement: Multimedia Appendix 1 [file nursing-v8-e75080-s001.pdf]

Hi ,

Are you interested in joining a research study titled "**Equitable Standards of Care Delivery in Team-based Risk Stratification of African Americans with Congestive Heart Failure".** If so, please complete the survey at the link below which should take about 15 minutes.

We are asking you to choose whether or not to join a research study about your beliefs and experiences regarding your use of the Electronic Medical Record, care delivery teams, and attitude toward it with respect improve the outcomes of African American patients with congestive heart failure. We want to learn about the best ways to assist nurses with the technology and improve the technologies for nurses. This gives you information to help you decide whether to join the study.

IF I JOIN THE STUDY, WHAT WILL I BE ASKED TO DO?

- Participate in a 15-minute online survey.

WHO MIGHT SEE THE INFORMATION YOU COLLECT ABOUT ME?
Only the study team will see the raw data. However, there are people who make sure the study is run the right way. These people may see identifying information. They are OHRP (Office for Human Research Protections), a federal agency, UAMS Institutional Review Board, and other institutional oversight offices. Because the study involves employees being asked about their perspectives and experiences about UAMS processes, their individual responses will not be shared outside the study team, and neither their faculty, supervisors, nor hospital administration will be told they participated in the study, nor given their individual responses.

CAN ANYTHING GOOD OR BAD HAPPENS TO ME IF I JOIN THE STUDY?
There will be no direct benefits to the study participants. We don't expect any bad things to happen to you. With any research, there is always a risk that someone may find out you were in the research and learn things about you that you don't want them to know. A risk to study participants is the potential for loss of confidentiality of study data. However, this risk is minimal due to the measures we will use to protect the confidentiality of study data. We will take the following steps to help prevent this from happening:

- No health information will be collected. The study team used your name and email address to contact you. However, your name and email address will not be maintained and will be replaced with a random number upon completion of this survey. Your name and email address will be destroyed on June 30, 2025.
- Your responses will be aggregated will all others.
- We will store the aggregated responses in a password-protected electronic format on a UAMS server for 7 years after the survey closes on June 30, 2025.

WILL I BE PAID FOR THIS STUDY?
There is a $25 incentive for participating in this survey. Immediately after participating in the survey, you will be emailed the incentive. If you get more than $600 in one year (January-December) from UAMS, UAMS may send you a tax form if the law requires it.

DO I HAVE TO TAKE PART IN THE STUDY?
No. It's important to remember that it's entirely up to you whether or not to join the study. Your participation in this survey is voluntary and nothing about your medical care or employee status will change as a result of whatever decision you make to join this study. Joining this survey is voluntary and not required but the information you provide would be valuable to the research.

CONTACT
If you have questions at any time about the study or the procedures, you may contact Tremaine Williams via email at tbwilliams@uams.edu. You may also contact the UAMS IRB at IRB@uams.edu or (501) 686-5667.

ELECTRONIC CONSENT: By clicking the survey link below, you agree to the following:

- You have read the above information.
- You voluntarily agree to participate in the study.
- You are age 18 or older.
