# Supplementary material for: Sociotechnical Needs of Registered Nurses in the Heart Failure Hospitalizations of African American Patients: Cross-Sectional Study
Source: JMIR Nurs. 2025 Dec 12;8:e75080. doi: 10.2196/75080 (PMC12700336; doi:10.2196/75080)
Supplement: Multimedia Appendix 2 [file nursing-v8-e75080-s002.pdf]

NURSING SURVEY OF EHR USE IN CHF PATIENTS

As a registered nurse, we are asking you to reflect on your beliefs and the experiences you've had with the electronic health record (EHR) throughout your career and the care you've provided with respect to improving the outcomes of African American patients with congestive heart failure (CHF). This survey is not specific to any one particular EHR system or institution you've been employed by.

We want to learn about the best ways to assist you with technology and improve the technologies for you.

Thank you for completing the questions below.

JOB TITLE: ______________________________

SEX:
- ◯ Male
- ◯ Female
- ◯ Unknown / Prefer Not to Answer

ETHNICITY:
- ◯ Hispanic or Latino
- ◯ Not Hispanic or Latino
- ◯ Unknown / Prefer Not to Answer

RACE:
- ◯ White
- ◯ Black or African American
- ◯ American Indian or Alaskan Native
- ◯ Asian
- ◯ Native Hawaiian or Other Pacific Islander
- ◯ Other Race
- ◯ Unknown / Prefer Not to Answer

Number of years working as a registered nurse (RN): ______________________________

FOR RNS ONLY - Number of years working with patients who have chronic illnesses: ______________________________

Number of years working as a licensed practical nurse (LPN): ______________________________

Number of years working in healthcare (including as a patient care tech, LPN, RN, or other role): ______________________________

Below you will see a range of tasks in sections A-D that you may undertake that are related to how you, as a registered nurse, work with the EHR to provide care for patients with congestive heart failure (CHF). Rate each of the tasks below based on the following:

• A) How important is the task to your work as an RN.

• B) How well you currently perform the task.

• C) I believe that additional changes in my work environment will improve my performance of the task.

• D) I believe that additional training, alone, will improve my performance of the task.

NOTE:

- For questions related to importance, use a scale ranging from 1 (Not at all Important) to 7 (Very Important).
- For performance-related questions, use a scale ranging from 1 (Not well) to 7 (Very well).
- For questions about beliefs regarding improvement, use a scale ranging from 1 (Strongly Disagree) to 7 (Strongly Agree).

1. Using the EHR to establish a relationship with African American patients who have CHF.

Note:

- **For questions related to importance, use a scale ranging from 1 (Not at all Important) to 7 (Very Important).**
- **For performance-related questions, use a scale ranging from 1 (Not well) to 7 (Very well).**
- **For questions about beliefs regarding improvement, use a scale ranging from 1 (Strongly Disagree) to 7 (Strongly Agree).**

	1	2	3	4	5	6	7
A) How important is this task is to your work as an RN	○	○	○	○	○	○	○
B) How well you currently perform this task	○	○	○	○	○	○	○
C) I believe that additional changes in my work environment will improve my performance of this task	○	○	○	○	○	○	○
D) I believe that additional training, alone, will improve my performance of this task	○	○	○	○	○	○	○

2. Reading published research on African Americans with CHF.

Note:

- **For questions related to importance, use a scale ranging from 1 (Not at all Important) to 7 (Very Important).**

- **For performance-related questions, use a scale ranging from 1 (Not well) to 7 (Very well).**

- **For questions about beliefs regarding improvement, use a scale ranging from 1 (Strongly Disagree) to 7 (Strongly Agree).**

	1	2	3	4	5	6	7
A) How important is this task is to your work as an RN	○	○	○	○	○	○	○
B) How well you currently perform this task	○	○	○	○	○	○	○
C) I believe that additional changes in my work environment will improve my performance of this task	○	○	○	○	○	○	○
D) I believe that additional training, alone, will improve my performance of this task	○	○	○	○	○	○	○

3. Caring for patients with CHF.

Note:

- **For questions related to importance, use a scale ranging from 1 (Not at all Important) to 7 (Very Important).**

- **For performance-related questions, use a scale ranging from 1 (Not well) to 7 (Very well).**

- **For questions about beliefs regarding improvement, use a scale ranging from 1 (Strongly Disagree) to 7 (Strongly Agree).**

	1	2	3	4	5	6	7
A) How important is the task is to your work as an RN	○	○	○	○	○	○	○
B) How well you currently perform this task	○	○	○	○	○	○	○
C) I believe that additional changes in my work environment will improve my performance of this task	○	○	○	○	○	○	○
D) I believe that additional training, alone, will improve my performance of this task	○	○	○	○	○	○	○

4. Caring for African American patients with CHF.

Note:

- **For questions related to importance, use a scale ranging from 1 (Not at all Important) to 7 (Very Important).**
- **For performance-related questions, use a scale ranging from 1 (Not well) to 7 (Very well).**
- **For questions about beliefs regarding improvement, use a scale ranging from 1 (Strongly Disagree) to 7 (Strongly Agree).**

	1	2	3	4	5	6	7
A) How important is the task is to your work as an RN	○	○	○	○	○	○	○
B) How well you currently perform this task	○	○	○	○	○	○	○
C) I believe that additional changes in my work environment will improve my performance of this task	○	○	○	○	○	○	○
D) I believe that additional training, alone, will improve my performance of this task	○	○	○	○	○	○	○

5. Giving patient education information to African American patients or their caregivers.

Note:

- **For questions related to importance, use a scale ranging from 1 (Not at all Important) to 7 (Very Important).**
- **For performance-related questions, use a scale ranging from 1 (Not well) to 7 (Very well).**
- **For questions about beliefs regarding improvement, use a scale ranging from 1 (Strongly Disagree) to 7 (Strongly Agree).**

	1	2	3	4	5	6	7
A) How important is the task is to your work as an RN	○	○	○	○	○	○	○
B) How well you currently perform this task	○	○	○	○	○	○	○
C) I believe that additional changes in my work environment will improve my performance of this task	○	○	○	○	○	○	○
D) I believe that additional training, alone, will improve my performance of this task	○	○	○	○	○	○	○

6. Drawing your own conclusions about how to use the EHR to care for African American patients.

Note:

- **For questions related to importance, use a scale ranging from 1 (Not at all Important) to 7 (Very Important).**

- **For performance-related questions, use a scale ranging from 1 (Not well) to 7 (Very well).**

- **For questions about beliefs regarding improvement, use a scale ranging from 1 (Strongly Disagree) to 7 (Strongly Agree).**

	1	2	3	4	5	6	7
A) How important is the task is to your work as an RN	○	○	○	○	○	○	○
B) How well you currently perform this task	○	○	○	○	○	○	○
C) I believe that additional changes in my work environment will improve my performance of this task	○	○	○	○	○	○	○
D) I believe that additional training, alone, will improve my performance of this task	○	○	○	○	○	○	○

7. Using risk scores or other information from the EHR to improve a patient's health.

Note:

- **For questions related to importance, use a scale ranging from 1 (Not at all Important) to 7 (Very Important).**

- **For performance-related questions, use a scale ranging from 1 (Not well) to 7 (Very well).**

- **For questions about beliefs regarding improvement, use a scale ranging from 1 (Strongly Disagree) to 7 (Strongly Agree).**

	1	2	3	4	5	6	7
A) How important is the task is to your work as an RN	◯	◯	◯	◯	◯	◯	◯
B) How well you currently perform this task	◯	◯	◯	◯	◯	◯	◯
C) I believe that additional changes in my work environment will improve my performance of this task	◯	◯	◯	◯	◯	◯	◯
D) I believe that additional training, alone, will improve my performance of this task	◯	◯	◯	◯	◯	◯	◯

8. Undertaking health promotion and prevention tasks to care for African American patients with CHF.

Note:

- **For questions related to importance, use a scale ranging from 1 (Not at all Important) to 7 (Very Important).**

- **For performance-related questions, use a scale ranging from 1 (Not well) to 7 (Very well).**

- **For questions about beliefs regarding improvement, use a scale ranging from 1 (Strongly Disagree) to 7 (Strongly Agree).**

	1	2	3	4	5	6	7
A) How important is the task is to your work as an RN	◯	◯	◯	◯	◯	◯	◯
B) How well you currently perform this task	◯	◯	◯	◯	◯	◯	◯
C) I believe that additional changes in my work environment will improve my performance of this task	◯	◯	◯	◯	◯	◯	◯
D) I believe that additional training, alone, will improve my performance of this task	◯	◯	◯	◯	◯	◯	◯

9. Assessing African American patients' clinical needs using the EHR.

Note:

- **For questions related to importance, use a scale ranging from 1 (Not at all Important) to 7 (Very Important).**
- **For performance-related questions, use a scale ranging from 1 (Not well) to 7 (Very well).**
- **For questions about beliefs regarding improvement, use a scale ranging from 1 (Strongly Disagree) to 7 (Strongly Agree).**

	1	2	3	4	5	6	7
A) How important is the task is to your work as an RN	○	○	○	○	○	○	○
B) How well you currently perform this task	○	○	○	○	○	○	○
C) I believe that additional changes in my work environment will improve my performance of this task	○	○	○	○	○	○	○
D) I believe that additional training, alone, will improve my performance of this task	○	○	○	○	○	○	○

10. Collecting relevant information on the social determinants of health (ex. education, health literacy, safe housing, access to nutritious food) from the EHR.

Note:

- **For questions related to importance, use a scale ranging from 1 (Not at all Important) to 7 (Very Important).**
- **For performance-related questions, use a scale ranging from 1 (Not well) to 7 (Very well).**
- **For questions about beliefs regarding improvement, use a scale ranging from 1 (Strongly Disagree) to 7 (Strongly Agree).**

	1	2	3	4	5	6	7
A) How important is the task is to your work as an RN	○	○	○	○	○	○	○
B) How well you currently perform this task	○	○	○	○	○	○	○
C) I believe that additional changes in my work environment will improve my performance of this task	○	○	○	○	○	○	○
D) I believe that additional training, alone, will improve my performance of this task	○	○	○	○	○	○	○

11. Working as a member of a CHF patient's care team.

Note:

- **For questions related to importance, use a scale ranging from 1 (Not at all Important) to 7 (Very Important).**
- **For performance-related questions, use a scale ranging from 1 (Not well) to 7 (Very well).**
- **For questions about beliefs regarding improvement, use a scale ranging from 1 (Strongly Disagree) to 7 (Strongly Agree).**

	1	2	3	4	5	6	7
A) How important is the task is to your work as an RN	○	○	○	○	○	○	○
B) How well you currently perform this task	○	○	○	○	○	○	○
C) I believe that additional changes in my work environment will improve my performance of this task	○	○	○	○	○	○	○
D) I believe that additional training, alone, will improve my performance of this task	○	○	○	○	○	○	○

12. Accessing clinical resources to care for your CHF patients.

Note:

- **For questions related to importance, use a scale ranging from 1 (Not at all Important) to 7 (Very Important).**

- **For performance-related questions, use a scale ranging from 1 (Not well) to 7 (Very well).**

- **For questions about beliefs regarding improvement, use a scale ranging from 1 (Strongly Disagree) to 7 (Strongly Agree).**

	1	2	3	4	5	6	7
A) How important is the task is to your work as an RN	○	○	○	○	○	○	○
B) How well you currently perform this task	○	○	○	○	○	○	○
C) I believe that additional changes in my work environment will improve my performance of this task	○	○	○	○	○	○	○
D) I believe that additional training, alone, will improve my performance of this task	○	○	○	○	○	○	○

13. Personally coping with burnout in your clinical environment.

Note:

- **For questions related to importance, use a scale ranging from 1 (Not at all Important) to 7 (Very Important).**
- **For performance-related questions, use a scale ranging from 1 (Not well) to 7 (Very well).**
- **For questions about beliefs regarding improvement, use a scale ranging from 1 (Strongly Disagree) to 7 (Strongly Agree).**

	1	2	3	4	5	6	7
A) How important is the task is to your work as an RN	○	○	○	○	○	○	○
B) How well you currently perform this task	○	○	○	○	○	○	○
C) I believe that additional changes in my work environment will improve my performance of this task	○	○	○	○	○	○	○
D) I believe that additional training, alone, will improve my performance of this task	○	○	○	○	○	○	○

14. Managing your overall workload of patients.

Note:

- **For questions related to importance, use a scale ranging from 1 (Not at all Important) to 7 (Very Important).**
- **For performance-related questions, use a scale ranging from 1 (Not well) to 7 (Very well).**
- **For questions about beliefs regarding improvement, use a scale ranging from 1 (Strongly Disagree) to 7 (Strongly Agree).**

	1	2	3	4	5	6	7
A) How important is the task is to your work as an RN	○	○	○	○	○	○	○
B) How well you currently perform this task	○	○	○	○	○	○	○
C) I believe that additional changes in my work environment will improve my performance of this task	○	○	○	○	○	○	○
D) I believe that additional training, alone, will improve my performance of this task	○	○	○	○	○	○	○

15. Working with hardware and software related to the EHR to care for a patient with CHF.

Note:

- **For questions related to importance, use a scale ranging from 1 (Not at all Important) to 7 (Very Important).**

- **For performance-related questions, use a scale ranging from 1 (Not well) to 7 (Very well).**

- **For questions about beliefs regarding improvement, use a scale ranging from 1 (Strongly Disagree) to 7 (Strongly Agree).**

	1	2	3	4	5	6	7
A) How important is the task is to your work as an RN	○	○	○	○	○	○	○
B) How well you currently perform this task	○	○	○	○	○	○	○
C) I believe that additional changes in my work environment will improve my performance of this task	○	○	○	○	○	○	○
D) I believe that additional training, alone, will improve my performance of this task	○	○	○	○	○	○	○

16. Working with information related to a patient's CHF in the EHR (ex. laboratory results, discharge summaries, or radiographic images) to care for the patient.

Note:

- **For questions related to importance, use a scale ranging from 1 (Not at all Important) to 7 (Very Important).**
- **For performance-related questions, use a scale ranging from 1 (Not well) to 7 (Very well).**
- **For questions about beliefs regarding improvement, use a scale ranging from 1 (Strongly Disagree) to 7 (Strongly Agree).**

	1	2	3	4	5	6	7
A) How important is the task is to your work as an RN	○	○	○	○	○	○	○
B) How well you currently perform this task	○	○	○	○	○	○	○
C) I believe that additional changes in my work environment will improve my performance of this task	○	○	○	○	○	○	○
D) I believe that additional training, alone, will improve my performance of this task	○	○	○	○	○	○	○

17. Working with the design of the EHR (ex. parts of the EHR's screens that you can see, touch, or hear) as you to retrieve information to provide care to patients with CHF.

Note:

- **For questions related to importance, use a scale ranging from 1 (Not at all Important) to 7 (Very Important).**
- **For performance-related questions, use a scale ranging from 1 (Not well) to 7 (Very well).**
- **For questions about beliefs regarding improvement, use a scale ranging from 1 (Strongly Disagree) to 7 (Strongly Agree).**

	1	2	3	4	5	6	7
A) How important is the task is to your work as an RN	○	○	○	○	○	○	○
B) How well you currently perform this task	○	○	○	○	○	○	○
C) I believe that additional changes in my work environment will improve my performance of this task	○	○	○	○	○	○	○
D) I believe that additional training, alone, will improve my performance of this task	○	○	○	○	○	○	○

18. The training or performance of other people in your environment who use the EHR (ex., other care team members, the EHR support team, CHF patients who interact with the EHR through patient portals).

Note:

- **For questions related to importance, use a scale ranging from 1 (Not at all Important) to 7 (Very Important).**
- **For performance-related questions, use a scale ranging from 1 (Not well) to 7 (Very well).**
- **For questions about beliefs regarding improvement, use a scale ranging from 1 (Strongly Disagree) to 7 (Strongly Agree).**

	1	2	3	4	5	6	7
A) How important is the task is to your work as an RN	○	○	○	○	○	○	○
B) How well you currently perform this task	○	○	○	○	○	○	○
C) I believe that additional changes in my work environment will improve my performance of this task	○	○	○	○	○	○	○
D) I believe that additional training, alone, will improve my performance of this task	○	○	○	○	○	○	○

19. Using current processes to share information that provides each CHF patient with the care they need at the time they need it.

Note:

- **For questions related to importance, use a scale ranging from 1 (Not at all Important) to 7 (Very Important).**

- **For performance-related questions, use a scale ranging from 1 (Not well) to 7 (Very well).**

- **For questions about beliefs regarding improvement, use a scale ranging from 1 (Strongly Disagree) to 7 (Strongly Agree).**

	1	2	3	4	5	6	7
A) How important is the task is to your work as an RN	○	○	○	○	○	○	○
B) How well you currently perform this task	○	○	○	○	○	○	○
C) I believe that additional changes in my work environment will improve my performance of this task	○	○	○	○	○	○	○
D) I believe that additional training, alone, will improve my performance of this task	○	○	○	○	○	○	○

20. Working within internal organizational policies, procedures, and culture related to the EHR to care for patients with CHF.

Note:

- **For questions related to importance, use a scale ranging from 1 (Not at all Important) to 7 (Very Important).**

- **For performance-related questions, use a scale ranging from 1 (Not well) to 7 (Very well).**

- **For questions about beliefs regarding improvement, use a scale ranging from 1 (Strongly Disagree) to 7 (Strongly Agree).**

	1	2	3	4	5	6	7
A) How important is the task is to your work as an RN	○	○	○	○	○	○	○
B) How well you currently perform this task	○	○	○	○	○	○	○
C) I believe that additional changes in my work environment will improve my performance of this task	○	○	○	○	○	○	○
D) I believe that additional training, alone, will improve my performance of this task	○	○	○	○	○	○	○

21. Working with external laws, regulations, and requirements that constrain your ability to use the EHR to prevent a CHF patient's death.

Note:

- **For questions related to importance, use a scale ranging from 1 (Not at all Important) to 7 (Very Important).**
- **For performance-related questions, use a scale ranging from 1 (Not well) to 7 (Very well).**
- **For questions about beliefs regarding improvement, use a scale ranging from 1 (Strongly Disagree) to 7 (Strongly Agree).**

	1	2	3	4	5	6	7
A) How important is the task is to your work as an RN	○	○	○	○	○	○	○
B) How well you currently perform this task	○	○	○	○	○	○	○
C) I believe that additional changes in my work environment will improve my performance of this task	○	○	○	○	○	○	○
D) I believe that additional training, alone, will improve my performance of this task	○	○	○	○	○	○	○

22. Continuously evaluate the quality of care that results from your use of the EHR to provide care for patients with CHF.

Note:

- **For questions related to importance, use a scale ranging from 1 (Not at all Important) to 7 (Very Important).**

- **For performance-related questions, use a scale ranging from 1 (Not well) to 7 (Very well).**

- **For questions about beliefs regarding improvement, use a scale ranging from 1 (Strongly Disagree) to 7 (Strongly Agree).**

	1	2	3	4	5	6	7
A) How important is the task is to your work as an RN	◯	◯	◯	◯	◯	◯	◯
B) How well you currently perform this task	◯	◯	◯	◯	◯	◯	◯
C) I believe that additional changes in my work environment will improve my performance of this task	◯	◯	◯	◯	◯	◯	◯
D) I believe that additional training, alone, will improve my performance of this task	◯	◯	◯	◯	◯	◯	◯
