# Supplementary material for: Sociotechnical Needs of Registered Nurses in the Heart Failure Hospitalizations of African American Patients: Cross-Sectional Study
Source: JMIR Nurs. 2025 Dec 12;8:e75080. doi: 10.2196/75080 (PMC12700336; doi:10.2196/75080)
Supplement: Multimedia Appendix 3 [file nursing-v8-e75080-s003.pdf]

Table S1. Importance and performance of sociotechnical tasks by group.

Item #	Socio-technical Task	Task Importance (Rating A)		Task Performance (Rating B)	
		Group A Mean (SD)	Group B Mean (SD)	Group A Mean (SD)	Group B Mean (SD)
**Adapted survey tasks**					
**1**	Using the EHR to establish a relationship with African American patients who have CHF	5.40 (1.68)	5.33 (1.82)	5.51 (1.53)	5.34 (1.49)
**2**	Reading published research on African Americans with CHF	4.89 (1.90)	4.88 (1.70)	3.90 (1.96)	3.83 (1.78)
**3**	Caring for all patients with CHF	5.93 (1.58)	6.21 (1.16)	5.52 (1.50)	5.86 (1.04)
**4**	Caring for African American patients with CHF	5.89 (1.64)	6.21 (1.30)	5.61 (1.44)	5.85 (1.26)
**5**	Giving patient education information to African American patients or their caregivers	6.13 (1.49)	6.29 (1.16)	5.41 (1.59)	5.62 (1.33)
**6**	Drawing your own conclusions about how to use the EHR to care for African American patients	4.66 (1.82)	4.94 (1.61)	4.54 (1.64)	4.90 (1.55)
**7**	Using risk scores or other information from the EHR to improve a patient's health	5.51 (1.62)	5.85 (1.32)	4.87 (1.66)	5.32 (1.38)
**8**	Undertaking health promotion and prevention tasks to care for African American patients with CHF	5.62 (1.70)	5.69 (1.67)	4.82 (1.57)	5.02 (1.56)
**9**	Assessing African American patients' clinical needs using the EHR	5.65 (1.63)	5.91 (1.34)	5.08 (1.55)	5.37 (1.38)
**10**	Collecting relevant information on the social determinants of health (ex. education, health literacy, safe housing, access to nutritious food) from the EHR.	5.85 (1.49)	5.83 (1.41)	5.07 (1.56)	5.29 (1.39)
**11**	Working as a member of a CHF patient's care team	5.68 (1.76)	5.88 (1.48)	5.19 (1.68)	5.67 (1.16)
**12**	Accessing clinical resources to care for your CHF patients	5.60 (1.70)	5.73 (1.51)	4.75 (1.70)	5.12 (1.40)
**13**	Personally coping with burnout in your clinical environment	6.19 (1.19)	6.23 (1.23)	4.42 (1.77)	4.50 (1.70)

**14**	Managing your overall workload of patients with the EHR	6.36 (1.17)	6.63 (0.71)	5.25 (1.45)	5.64 (1.20)
**Sittig and Singh's tasks**					
**15**	Working with hardware and software related to the EHR to care for a patient with CHF	5.32 (1.82)	5.62 (1.60)	4.78 (1.60)	5.19 (1.40)
**16**	Working with information related to a patient's CHF in the EHR (ex. laboratory results, discharge summaries, or radiographic images) to care for the patient	5.76 (1.72)	6.19 (1.29)	5.36 (1.53)	5.80 (1.26)
**17**	Working with the design of the EHR (ex. parts of the EHR's screens that you can see, touch, or hear) as you retrieve information to provide care to patients with CHF	5.31 (1.92)	5.73 (1.42)	4.72 (1.62)	5.33 (1.29)
**18**	The training or performance of other people in your environment who use the EHR (ex., other care team members, the EHR support team, and CHF patients who interact with the EHR through patient portals).	5.33 (1.83)	5.51 (1.51)	4.77 (1.62)	5.04 (1.43)
**19**	Using current processes to share information that provides each CHF patient with the care they need at the time they need it	5.52 (1.81)	5.75 (1.45)	4.80 (1.62)	5.24 (1.32)
**20**	Working within internal organizational policies, procedures, and culture related to the EHR to care for patients with CHF	5.49 (1.78)	5.56 (1.55)	4.75 (1.57)	5.06 (1.44)
**21**	Working with external laws, regulations, and requirements that constrain your ability to use the EHR to prevent a CHF patient's death	4.93 (2.04)	5.21 (1.88)	4.38 (1.79)	4.75 (1.67)
**22**	Continuously evaluate the quality of care that results from your use of the EHR to provide care for patients with CHF	5.26 (1.85)	5.63 (1.60)	4.66 (1.72)	5.08 (1.68)

Table S2. Significant sociotechnical needs by group.

Item #	Group A				Group B			
	Mean Difference (A – B)	Cohen's d (95%, CI)	*P*-value	FDR *P*-value	Mean Difference (A – B)	Cohen's d (95%, CI)	*P*-value	FDR *P*-value
**Adapted survey tasks**								
**1**	-0.11	-0.07 (-0.35,0.21)	.63	.63	-0.01	-0.01 (-0.28,0.27)	.97	.97
**2**	0.99	0.51 (0.23,0.79)[b]	<.001	<.001	1.05	0.60 (0.32,0.89)[b]	<.001	<.001
**3**	0.41	0.27 (-0.01,0.54)	.06	.08	0.35	0.32 (0.04,0.60)[a]	.03	.04
**4**	0.28	0.18 (-0.1,0.46)	.20	.22	0.36	0.28 (0,0.56)	.05	.06
**5**	0.72	0.47 (0.19,0.75)[a]	.001	.003	0.67	0.54 (0.25,0.82)[b]	<.001	<.001
**6**	0.12	0.07 (-0.21,0.35)	.63	.63	0.04	0.03 (-0.25,0.30)	.86	.90
**7**	0.64	0.39 (0.11,0.67)[a]	.006	.01	0.53	0.39 (0.11,0.67)[a]	.006	.02
**8**	0.80	0.49 (0.21,0.77)[a]	.001	.003	0.67	0.42 (0.13,0.69)[a]	.004	.02
**9**	0.57	0.36 (0.08,0.64)[a]	.01	.02	0.54	0.40 (0.12,0.68)[a]	.006	.02
**10**	0.78	0.51 (0.23,0.79)[b]	<.001	<.001	0.54	0.38 (0.1,0.66)[b]	.007	.02
**11**	0.49	0.29 (0.01,0.56)	.045	.06	0.21	0.16 (-0.12,0.44)	.27	.29
**12**	0.85	0.50 (0.22,0.78)[b]	.001	.003	0.61	0.42 (0.14,0.70)[a]	.003	.01

**13**	1.77	1.17 (0.87,1.47)[c]	<.001	<.001	1.73	1.17 (0.87,1.47)[c]	<.001	<.001
**14**	1.11	0.84 (0.55,1.13)[c]	<.001	<.001	0.99	1.00 (0.71,1.30)[c]	<.001	<.001
**Sittig and Singh's tasks**								
**15**	0.54	0.32 (0.04,0.59)[a]	.03	.04	0.43	0.29 (0.01,0.56)	.045	.06
**16**	0.40	0.25 (-0.03,0.52)	.08	1	0.39	0.31 (0.03,0.58)[a]	.03	.047
**17**	0.59	0.33 (0.05,0.61)[a]	.02	.03	0.40	0.30 (0.02,0.57)	.04	.052
**18**	0.56	0.32 (0.04,0.60)[a]	.023	.04	0.47	0.32 (0.04,0.60)[a]	.03	.04
**19**	0.72	0.42 (0.14,0.70)[a]	.003	.007	0.51	0.37 (0.09,0.65)[a]	.01	.02
**20**	0.74	0.44 (0.16,0.72)[a]	.002	.005	0.50	0.33 (0.05,0.61)[a]	.02	.04
**21**	0.55	0.29 (0.01,0.57)	.63	.06	0.46	0.26 (-0.02,0.54)	.07	.08
**22**	0.60	0.34 (0.06,0.62)[a]	<.001	.03	0.55	0.34 (0.06,0.61)[a]	.02	.04

[a]Small Effect Size
[b]Moderate Effect Size
[c]Large Effect Size
