# Supplementary material for: Sociotechnical Needs of Registered Nurses in the Heart Failure Hospitalizations of African American Patients: Cross-Sectional Study
Source: JMIR Nurs. 2025 Dec 12;8:e75080. doi: 10.2196/75080 (PMC12700336; doi:10.2196/75080)
Supplement: Checklist 1 [file nursing-v8-e75080-s004.pdf]

Checklist for Reporting Results of Internet E-Surveys (CHERRIES)

Item category	Checklist item	Your Study
**Design**	Describe survey design	This was a single-site study that employed an observational, cross-sectional survey design. The observational nature of the study utilized EHR data from the Arkansas Clinical Data Repository to retrospectively track a target population of 3,498 RNs who provided care to 22,703 patients with CHF within 113,543 heart failure hospitalizations between January 1, 2015, and January 1, 2024. The repository houses data extracted from the EPIC EHR and legacy systems and has been a secure source of data for other clinical and translational studies of the population. [30] Two hundred RNs were randomly selected using stratified random sampling based on the mean length of stay of their African American patients. An adapted version of the Hennesey-Hicks Needs Analysis survey was administered to all RNs in the sample. (Line 168-180)
**IRB approval and informed consent process**	IRB approval	The study procedures (Protocol #276211) were reviewed and approved by the Institutional Review Board. (Line 183-184)
	Informed consent	The informed consent document embedded within the email included a basic description of the purpose of the study, the anticipated length of time survey completion, details on how data would be stored and accessed (where, how long), and contact information for the principal investigator. After reviewing and agreeing to the informed consent presented via email, RNs were routed to the survey. A waiver of documentation of the informed consent process was granted by the Institutional Review Board because the study presented only minimal risk for loss of confidentiality. (Line 189-199)
**Data protection**		Data protection processes included not sharing individual responses outside of the study team, aggregating responses,

		segmenting access to identifying data and responses within the study team, storing data on only one University-encrypted, laptop with multiple firewalls, and deleting identifying information after the study closed on June 30, 2025. (Line 192-199)
**Development and pre-testing**	Survey development	The survey, endorsed by the World Health Organization, is globally used within healthcare to assess task importance, performance, and needs of RNs and other clinicians. [27] Thirty-three prior studies of clinical practice have demonstrated the survey's translation, cultural adaptation, and psychometric reliability in revealing the needs of RNs and other clinicians. [27] The standard survey contained 30 items consisting of basic care tasks. [30] After adaptation, a total of 22 items were in the final survey and represented the socio-technical tasks performed by RNs during the provision of care. The survey instrument is provided in Multimedia Appendix 2.<br><br>Of the 22 final items, Item's 1-14 were adapted from the basic items on the standard survey. The items were modified in alignment with the psychometric requirements of the survey manual. [27] For example, Item #1 of the standard survey required RNs to rate the following statement: "Establishing a relationship with patients". The item was adapted for this study to state, "Using the EHR to establish a relationship with African American patients who have CHF." The survey allowed for the removal and substitution of eight of the standard items without compromising the psychometric properties (i.e., reliability, validity) of the instrument. Eight of the standard survey items were removed because they did not align with the objective of the study.

		The remaining items, Item's 15-22, were directly adapted from all eight dimensions of the study's conceptual framework, the Sittig and Singh's Socio-technical Framework for Health Information Technology (Table 1). [26] The framework was chosen because it is the most comprehensive framework for understanding how RNs work with EHRs to improve patient outcomes. (Line 202-223).
	Testing	The survey was pilot tested for usability and technical functionality among an independent group of five RNs who reflected the sampling frame of the target population. The changes included increasing reminder intervals from monthly to bi-weekly, condensing introductory text for questions, and removing duplicate demographics data collection. (Line 229-232)
**Recruitment process and sample**	Open survey vs closed survey	Closed survey. Only pre-identified RNs were surveyed. Using a closed survey approach, all 200 RNs completed an electronic version of the same survey through the University-based, Research Electronic Data Capture System. [36-37] Email addresses were loaded into the system and managed by one member of study team for automated bi-weekly survey reminders and to prevent multiple entries from the same individual. Each RN received a custom survey link which allowed for the study team to determine completion. (Line 277-281)
	Contact mode	Email only
	Advertising the survey	Not applicable.
**Survey administration**	Web/email	Emailed a web-based survey hosted by the University-based, Research Electronic Data Capture System.
	Context	Conducted in a hospital setting.
	Mandatory/voluntary	Participation was voluntary.

	Incentives	A $25 gift card was provided after survey completion.
	Time/Date	The survey was administered between November 15, 2024, and January 31, 2025.
**Randomization of items**		Items or questionnaires not randomized.
**Adaptive questioning**		Adaptive questioning not applied.
**Number of items**		The RNs manually rated all 22 survey items (socio-technical tasks) on a seven-point Likert scale in four categories (Rating A-D). (Line 288-289)
**Number of screens/pages**		The RNs rated approximately five survey items on each of the six pages of the web-based, survey in the Research Electronic Data Capture System. (Line 288-292)
**Completeness check**		Responses were reviewed for completnes immediately upon submission. There were no incomplete responses. (Line 365)
**Review step**	Participants could revise their responses before final submission.	The RNs were able to modify their ratings by toggling back and forth through the survey items before submission. (Line 288-292)
**Response rates**	Unique site visitor	Not applicable.
	View rate	Not applicable.
	Participation rate	Not applicable.
	Completion rate	All 200 RNs completed the survey (100% survey completion rate) and there was no attrition. (Line 365)
**Preventing multiple entries**	Cookies used	Not applicable.
	IP check	Not applicable.
	Log file analysis	Not applicable.
	Registration	Participants were identified through a data request to the Arkansas Clinical Data

		Warehouse based on electronic health records and invited emailed to participate in the survey. (Lines 168-172)
**Analysis**	Handling incomplete questionnaires	There were no incomplete questionnaires.
	Atypical timestamps	Not applicable.
	Statistical correction	Descriptive statistics, difference score, Cohen's d coefficient, and a two-sided unpaired t-test were used to identify the perceived importance of these socio-technical tasks, the perceived performance of these tasks by RNs, and their socio-technical needs in relation to the length of stay of their African American patients with CHF. (Line 168-180)
